# Single Nucleotide Polymorphisms as Practical Molecular Tools to Support European Chestnut Agrobiodiversity Management

**DOI:** 10.3390/ijms21134805

**Published:** 2020-07-07

**Authors:** Angelina Nunziata, Valentino Ruggieri, Milena Petriccione, Luigi De Masi

**Affiliations:** 1 C.R.E.A. Council for Agricultural Research and Economics—Research Centre for Olive, Fruits and Citrus Crops, Via Torrino 2, 81100 Caserta, Italy; milena.petriccione@crea.gov.it; 2 Biomeets Consulting, ITNIG—Carrer d’Àlaba, 6108005 Barcelona, Spain; valentino.ruggieri@biomeets.es; 3 National Research Council (CNR), Institute of Biosciences and BioResources (IBBR), Via Università 133, 80055 Portici (NA), Italy

**Keywords:** *Castanea sativa*, cultivar, molecular markers, DNA polymorphism, RAPD, EST, HRM, SNP, KASP

## Abstract

European chestnut orchards are multifunctional agroforestry systems with a key role in environmental management. Their biodiversity is at risk of erosion and farmers do not have enough tools to protect and valorize traditional ecotypes. In particular, cost effective and reliable molecular markers for cultivar identification are lacking. The aim of this research was to develop a new molecular tool for varietal identification in European chestnuts. A set of cultivars was preliminarily characterized to evaluate the range of genetic diversity using random amplified polymorphic DNA (RAPD) markers. The genetic distances indicated a sufficiently wide variability range among tested genotypes and confirmed they were suitable for our goal. A single nucleotide polymorphism (SNP) mining within 64 expressed sequence tags (EST), covering all the linkage groups, was performed by high-resolution melting (HRM) and validated by target resequencing. Fifty-six SNPs were retrieved by monitoring the variability present on the whole set of considered cultivars in loci uniformly distributed on the genome. A subset of 37 SNPs was finally transformed into kompetitive allele-specific PCR (KASP) markers that were successfully evaluated for varietal discrimination. Three assays (C1083, G0115 and A5096) were identified as necessary and sufficient for distinguishing among the tested cultivars. The developed tools can be effectively exploited by stakeholders for improving the management of the European chestnut genetic resources.

## 1. Introduction

In 2018, chestnut production worldwide was above 2.3 million tons. China is the world’s biggest producer of chestnuts accounting for about 83.4%, while Italy ranks among the top five producing countries [1]. The genus *Castanea* includes up to 13 species and belongs to the family Fagaceae, which also includes, among others, the genera *Quercus* and *Fagus* [2]. European or sweet chestnut (*Castanea sativa* Mill.), Japanese chestnut (*Castanea crenata* Sieb. et Zucc.), Chinese chestnut (*Castanea mollissima* Bl.) and American chestnut (*Castanea dentata* Borkh.) are farmed in temperate areas of Europe/Asia Minor, Japan/the Korean peninsula, China and North America, respectively. However, in recent years, many interspecific hybrids were used for fruit and timber production or as rootstock [3].

European chestnut grows in high forests, simple coppices or grafted orchards, representing historical and cultural heritage in hills and mountainous areas. Chestnut orchards are multifunctional agroforestry systems that provide, among other things, timber and edible fruits [4,5,6]. Its cultivation characterizes hilly landscapes and medium-height mountains, areas with a high risk of fire and erosion, where the growers are responsible for territory overseeing. Nowadays, in the Southern regions of Italy, European chestnut is a threatened fruit tree species, but the long tradition of chestnut cultivation has allowed the spread of several traditional varieties and many of them are still sold in the local markets [7,8]. Campania Region is a leader for chestnut production, with a cultivation area of 13,800 ha, followed by other Regions such as Tuscany (10,400 ha), Calabria (8600 ha), Piedmont (6400 ha) and Lazio (3800 ha) [4]. Approximately 12% of the total Italian chestnut production is represented by marrone-type cultivars, while the other 88% is constituted by other *C. sativa* cultivars and Euro-Japanese hybrids. Italian production is commercialized in local markets or exported as fresh chestnuts (75%), processed food (20%) and animal food (5%) [4]. The Campania Region represents an important reservoir of valuable chestnut genotypes, which were developed over the years for spontaneous hybridization and subsequent selections made by the farmers to obtain genotypes adapted to local conditions and with quality requirements well delineated for specific uses. In the Regional Park of “Roccamonfina–Foce Garigliano”, the local farmers have selected and preserved different genotypes contributing to the conservation and maintenance of agrobiodiversity of the chestnut germplasm so that, until now, an important part of chestnut genetic diversity has been hosted in this small area [8]. Many local cultivars received great attention in supporting the social, historical, and cultural identity of old and remote rural centers. The molecular characterization of genetic resources is needed to preserve agrobiodiversity, especially where old growers, experts in the morphological discrimination of local cultivars, are being substituted by younger ones. Assessing the genetic diversity of local genotypes, in fact, could help in limiting genetic erosion and safeguarding this important genetic heritage.

If compared to the relative significance of the species within the genus, genomic resources for *C. sativa* are limited. In fact, the relative economic importance of *C. sativa* within the genus can be estimated considering that the production of chestnut fruits in the areas of interest for this cultivation in 2018, namely Europe and Asia Minor, totaled 218,806 Mg (Europe—154,612 Mg, Turkey—63,580 Mg, Azerbaijan—614 Mg), about 9% of the 2,353,825 Mg of chestnuts produced all over the world [1]. This percentage was even higher in 1997 (25%) due to a decrease of 64% in European chestnut production [1]. Moreover, in order to estimate the relative ecological importance of the species within the genus, it has to be considered that the area of interest for *C. sativa* cultivation in Europe and Asia Minor in 2018 was 158,866 ha (Europe—119,494 ha, Turkey—39,080 ha, Azerbaijan—292 ha) about 26% of the 612,877 ha of chestnuts cultivated all over the world [1]. Despite this economic and ecological relative impact of 9% and 26%, respectively, to date, only 1% of the sequences in the National Center for Biotechnology Information (NCBI) nucleotide databases for the genus *Castanea* refer to the species *C. sativa* (854 sequences out of 75,760) and <1% of Next Generation Sequencing (NGS) datasets (five out of 2900) [9]. Beyond these genomic data, two other databases including 8217 and 6819 contigs from two *C. sativa* samples can be retrieved in the Costa Project [10], aiming to gain knowledge of molecular mechanisms involved in tolerance to oomycete *Phytophthora cinnamomi* [11,12]. These two datasets were generated by sequencing root transcriptomes of European chestnut (*C. sativa*, susceptible to the pathogen) during programmed conditions of infection. Nonetheless, these data are not fully annotated and are not available for BLAST analyses online. Furthermore, the first reference unigene catalog for the European chestnut in two chestnut cultivars (cynipid resistant and cynipid susceptible) has only recently been realized [13].

Despite this limited genomic data availability, many molecular markers for European chestnut selection and genetic characterization have been developed in the past 20 years. Among these, the first used in a linkage map for European chestnut were random amplified polymorphic DNA (RAPD), inter simple sequence repeat (ISSR) and isozyme markers [14]. While isozymes and ISSR have been progressively abandoned, RAPDs are still in use for genetic diversity screening due to their cost effectiveness and easy-to-use application. RAPD markers were particularly useful to detect DNA variations (polymorphisms) without knowledge of the genome sequence. Nonetheless, they require a very standardized procedure to be transferable from one laboratory to another. At present, there are only a few studies on the characterization of the Roccamonfina chestnuts by using pheno-morphological parameters and DNA-based markers [7,15,16]. In particular, Galderisi et al. [7] successfully employed RAPD markers for genotyping, among others, some local chestnut cultivars of Roccamonfina (‘Mercogliana’, ‘Napoletana’, and ‘Tempestiva’). In subsequent years, microsatellites (or simple sequence repeats; SSR) were developed, often deriving from ISSR sequencing, and 70 microsatellite sequences were annotated in NCBI for *C. sativa* [17,18,19]. These markers are efficient, reliable and were widely used for population studies and cultivar genotyping. Recently, they have been used for characterizing Iberian and British populations and, combined with morphological observations, they allowed to come to important practical conclusions about natural populations’ biodiversity [20,21]. Nonetheless, they require a workflow that is not compatible with cost effective and easy-to-use applications as it includes a thorough DNA purification, amplification in presence of fluorescent-labeled primers, capillary electrophoresis, as well as complex data elaboration. Recent studies on Japanese and European chestnuts evidenced that molecular marker-assisted breeding, as well as other field applications, better benefits from high-throughput markers based on single nucleotide polymorphisms (SNPs) [13,22,23]. Nonetheless, the markers developed in the cited studies were mined within *C. crenata* and interspecific *C. crenata* × *C. sativa* breeding progenies and SNPs specific for *C. sativa* are still lacking. This kind of marker is generally developed by starting from a comparison between two genotypes, even if recent SNP arrays came from the comparison of eight [24] to 27 [25] accurately chosen genotypes. The inclusion of many genotypes aims at building a set of markers with an a priori maximization of marker performances. Alongside these, other approaches for a high-throughput screening of genetic variability on a smallest number of loci rely on detection of polymorphism by high-resolution melting (HRM) analysis followed by punctual resequencing [26,27]. Mined mutant alleles can then be screened by HRM or converted in other more practical and user-friendly tests for SNP detection. Kompetitive allele-specific PCR (KASP) is a smart method that includes the same advantages of genotyping based on allele-specific fluorescence resonance energy transfer (FRET) endpoint PCR (TaqMan™ like) in terms of simple workflow and complete scalability, but with a much cheaper assay production and validation [28].

The main aim of this work was to develop a practical molecular tool for varietal identification in European chestnuts. Therefore, (i) the genetic diversity of a local pool of chestnut varieties was assessed by RAPD markers to verify whether it was suitable for implementing the innovative tools, (ii) new SNPs were identified through HRM analysis followed by resequencing and (iii) identified SNPs were transformed in an effective KASP assay.

## 2. Results

### 2.1. Preliminary Assessment of Genetic Diversity in Chestnut Cultivars by RAPD Molecular Markers

**Figure 1:**
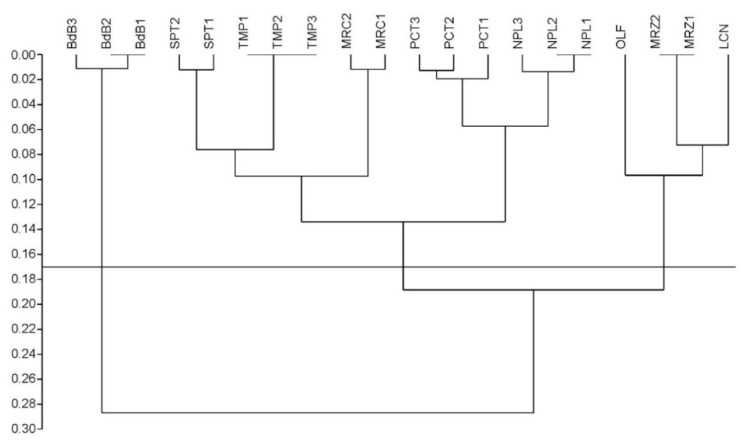
Clustering of the nine chestnut cultivars under investigation created by unweighted pair group method with arithmetic mean (UPGMA) analysis of random amplified polymorphic DNA (RAPD) data. The numerical scale indicates the degree of genetic distance by Dice’s coefficient (Dc). The average Dc of 0.17 was used as cut-off line to detect the main clusters.

The performed preliminary genetic analysis allowed us to estimate the genetic variability among eight chestnut cultivars from Campania together with the hybrid ‘Bouche de Bétizac’ (BdB). Twenty arbitrary primers were screened; out of these, seven were selected on the basis of signal quality and reproducibility, and six of them showed DNA polymorphisms. The RAPD amplification results were reported in Table S1. The analysis of the chestnut RAPD bands identified a total of 60 markers or loci, the number detected for each chestnut DNA sample was dependent on the variety and primer used, ranging from three to 10 bands per primer with an average of 8.6 loci, for a total of 1200 data points. Of these markers, 39 loci were polymorphic (65%) and their variation made it possible to discriminate with certainty the eight varieties of *C. sativa* between them and the Euro-Japanese hybrid and, at the same time, determine their genetic relationships. Among the detected polymorphisms, 16 alleles turned out to be private, indicating that the relative bands were genotype-specific to a single clonal population. These markers are diagnostic in order to properly identify material from chestnut varieties. Although ‘Lucente’ (LCN), ‘Napoletana’ (NPL), and ‘Tempestiva’ (TMP) lacked private alleles, their DNA profiles allowed varietal discrimination as a result of cultivar-specific combinations of bands. RAPD markers clearly differentiated the hybrid genotype from the local chestnut cultivars. Their genetic relationships are shown in Table S2, where the genetic distances among all samples measured by Dice’s coefficient (Dc) varied from zero (for example, within the clones of TMP) to 0.34 between ‘Paccuta’ clone 1 (PCT1) and BdB3. The total average Dc of 0.17 is indicative of the average genetic distance. These distances corresponded to complementary similarity values between 0.66 and one, and revealed a correlation of 98.5%, with the normal distribution computed by a normal probability plot in PAST (PAleontological STatistics) [29] (plot not shown). As expected, the genetic distances registered between *C. sativa* cultivars showed a narrower range with a minimum of 0.04 between PCT1 and NPL1/NPL2, and a maximum of 0.27 between ‘Olefarella’ (OLF) and ‘San Pietro’ clone 1 (SPT1), corresponding to a similarity range distributed from 0.73 to 0.96. Then, the genetic distance matrix based on Dc (Table S2) was used to build a dendrogram, as shown in Figure 1.

The Cophenetic Correlation Coefficient (CP) of 0.93 expressed the high consistency of the whole dendrogram. A cluster analysis based on genetic polymorphisms confirmed the morphological classification. The dendrogram topology depicted the traditional cultivars grouped all together into two distinct and large clusters, also supporting their common origin, while the hybrid ‘Bouche de Bétizac’ was located apart, likely as an outgroup. A significantly low level of genetic distance, lower than the average Dc of 0.17, was evidenced for these main clusters, subdividing the cultivars into three groups: (A) ‘Mercogliana’ (MRC)–NPL–PCT–SPT–TMP, (B) LCN–MRZ–OLF, and (C) BdB. Although TMP was grouped together with traditional cultivars into cluster A of the RAPD-based dendrogram (Figure 1), this cultivar showed the least genetic distance with BdB (Table S2). Moreover, since we sampled from one to three trees per variety, in some cases trees belonging to the same variety showed minimal genetic differences at the clonal level, evidenced by sub-clustering, as in MRC, NPL, PCT, and SPT of cluster A (but not in TMP), and in BdB of cluster C, represented by itself.

### 2.2. SNP Mining in C. sativa

#### 2.2.1. Selection of Target Loci from *C. Crenata*

Candidate SNP have been selected within available *C. crenata* expressed sequence tags (EST) that had already proved to be polymorphic within this species [22]. Assuming a general synteny between *C. crenata* and *C. sativa* genomes [11,30], 64 loci were selected and uniformly distributed in available linkage maps of *C. crenata*, approximately 10 cM from each other. The chosen loci were coded by a letter indicating the linkage group followed by a number code related to the approximate distance from the telomere. On their sequences, primer pairs were designed to amplify 150 bp fragments so that the candidate SNPs were as much as possible in the center of the fragment. In fact, for subsequent test design, at least 50 bp flanking the SNP on each side have to be provided. Primer design allowed us to gain products with prefixed lengths in 61 on 64 cases, with the length of flanking sequences being shorter than 50 bp only in six of 128 flanking sequences distributed on five fragments. Primer sequences and the lengths of amplified fragments are provided in Table S3.

#### 2.2.2. High-Resolution Melting (HRM) Screening to Select Polymorphic *C. sativa* DNA Fragments for Subsequent Resequencing

**Figure 2:**
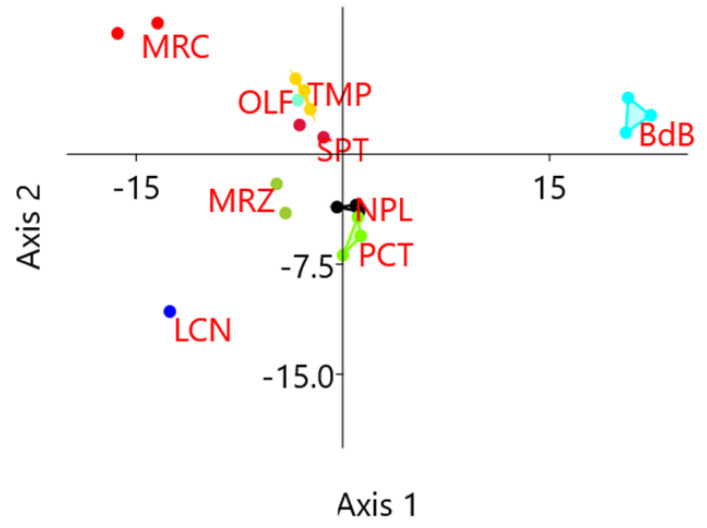
Results of high-resolution melting (HRM) analyses: coordinates on the first two canonical axes derived from linear discriminant analysis (LDA) computed on HRM diversity data. Genotypes from the same cultivar cluster together and can be discriminated from the others.

HRM reactions involved 64 loci and 20 chestnut DNA samples, for a total of 1280 HRM analyses. Among these, 1208 samples gave positive results so that HRM curves could be compared to each other. Cluster analyses were performed on normalized HRM curves in standard conditions and the results allowed for the identification of one to seven clusters of melting per locus, with an average of three cluster per locus (Table S4). HRM analyses identified six loci without polymorphisms, so no further analyses were performed on them: E3, E4, G2, H1, I1, and J3. These loci were scored as particularly conserved as they presented neither transferable inter-specific polymorphisms nor any other new SNPs. The other 58 analyzed loci, instead, were scored as candidate polymorphic traits as they presented more than one denaturing cluster. Within each locus, one amplified sample per cluster was selected for subsequent resequencing. HRM polymorphisms were also evaluated in terms of cultivar discrimination power by the unweighted pair group method with arithmetic mean (UPGMA) clustering, applying the Hamming index to results in Table S4 previously converted into a coded table (same color, same letter). Samples of the same cultivar mostly clustered together (Figure S1) with a bootstrap value higher than 78%. The distances between samples of the same cultivars ranged from 0.02 to 0.22, except for genotypes belonging to PCT that did not cluster together. It is worth noting that the genotypes PCT1 and PCT2 showed a high percentage of missing values (11% and 55%, respectively). Linear discriminant analysis (LDA) on the same results evidenced a confusion matrix with 95% of correctly classified genotypes, with only one sample of the group PCT being incorrectly predicted as belonging to group NPL (Table S5). In Figure 2, genotypes are represented using the first two canonical axes derived from LDA and convex hulls represent the smallest polygons containing all genotypes from the same cultivar. These results evidence that the polymorphism detected in 58 loci is sufficient to discriminate samples of different cultivars.

#### 2.2.3. Target Resequencing of Polymorphic Fragments Selected by HRM Analysis

HRM results allowed for the selection of 192 out of the 1280 screened fragments for subsequent resequencing. The fragments, covering two to five genotypes per locus, were resequenced to obtain a total of 8963 bp distributed in 58 loci, with an average sequencing coverage of 3.1×. Overall, a high sequence similarity was detected between *C. sativa* and *C. crenata*, with an average level of diversity comparable to that found between cultivars of *sativa* species.

Among the 58 resequenced loci, ten did not show the expected polymorphism, as sequencing of fragments from genotypes in different HRM clusters resulted in the same sequence. These ten loci, namely A2, A6, D0, D2, D4, F2, I1, I3, J3, and J5, were scored as non-polymorphic and the relative SNPs registered in *C. crenata* were scored as not transferable to *C. sativa*. The remaining 48 loci harbored 70 SNPs (Table S6), 37 being synonymous, 32 missense variants and just one mapping outside of the coding sequence (intergenic variant).

Out of the 70 SNPs, 18 overlap with those described by Nishio et al. [22]. Nonetheless, among these 18 inter-specific SNPs, 14 were private for ‘Bouche de Bétizac’, and only four were indeed useful to distinguish among the examined European chestnut cultivars. As result, 52 SNPs, never previously described in the *Castanea* genus, were identified. The total set of 70 SNPs identified is reported in Table 1 and Table S6. Alongside the functional description of the genes affected by the SNPs, Table 1 reports the nucleotide substitution in the context of the coding triplet, and its putative impact on the protein functionality. Supplementary Table S7 reports the gene ontology (GO) terms of the 48 selected loci. Since the SNPs were selected randomly across the genome, any enrichment in specific function was observed. However, excluding seven loci that did not show any functional description, about half of the remaining 41 loci showed molecular function GOs mainly annotated as binding activity (nucleic acid binding, metal ion binding). As for the biological process terms, GOs frequently referred to protein phosphorylation, protein imports into the nucleus, DNA repair and proteolysis. 

### 2.3. KASP Assay Selection for Varietal Identification

Among the 56 SNPs useful for cv. discrimination (four overlapping with Nishio et al. [22] and 52 discovered by the present resequencing), 37 were selected as having at least 50 described flanking bases on the right and left. Then, they were used for developing KASP assays and for validating the markers as a tool for cultivar discrimination. In particular, the four inter-specific transferable SNPs A9081, F1081, K0081, and L4081 were used together with 33 other newly identified SNPs. The 37 KASP assays monitored traits in different linkage groups, as identified by Nishio et al. [22], namely five in group A, five in group B, seven in group C, two in group D, three in group E, two in group F, three in group G, one in group H, three in group J, three in group K and three in group L, so that 11 of the 12 linkage groups were involved in the analyses. The results, shown in Table 2, evidenced that a unique data string can be written for genotypes belonging to the same cultivar, so that no cv. miscalling can occur.

**Figure 3:**
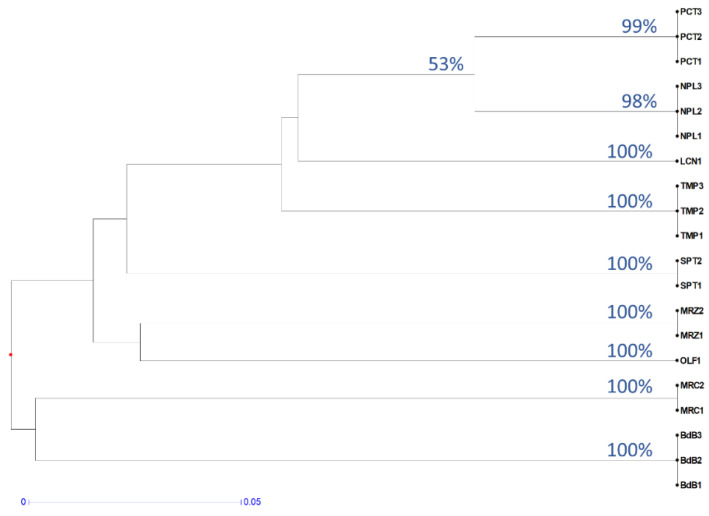
Dendrogram based on KASP results. UPGMA clustering was performed on distances computed by the simple matching index for diploid codominant marker. Bootstrap indices ≥53% are reported on the nodes. The distance is represented by the length of the branch according to the reported scale.

A cluster analysis of the KASP results confirms that the method is suitable for cv. identification (Figure 3). In fact, bootstrap values for each group of genotypes were 98–100% and the similarity between genotypes of the same cv. was 100%. The bootstrap between clades of different cultivars was, as expected, always lower than 60%. In fact, an analysis of the relationships between the different cultivars was out of the remit of these KASP tests and requires a major number of SNPs. The clade including more than one cultivar with the highest bootstrap value (namely 53%) was the one between PCT and NPL, which was also found by a cluster analysis of the RAPD data (Figure 1).

These data have also been screened for redundancy and, as expected, the minimum number of tests required for cv. identification can be considerably reduced. In particular, as a result of a KASP test is a letter code with three changes (SNP homozygous for allele 1, or for allele 2, or heterozygous), the minimum theoretical number of tests necessary to distinguish between nine different cultivars, should be 2 (3^2^ = 9), but this could be achieved only providing that the found SNPs had a minor allele frequency (MAF) of 0.5 and an observed heterozygosity of 0.33. Among the KASP assays with the highest value for minimum allele frequency, we were able to identify the three assays C1083, G0115 and A5096 as the necessary and sufficient number for distinguishing among the nine tested cultivars. The response codes, unique for each cultivar, are listed in Table 3.

## 3. Discussion

The conservation of the genetic materials, selected over centuries, is necessary for maintaining agrobiodiversity [8]. The huge varietal richness in the European chestnut germplasm is at high risk of genetic erosion due to the varietal substitution engaged to counter the adversities. Moreover, genotypes are multiplicated by clonal propagation techniques and the exchange of genetic materials has contributed to synonymy and homonymy issues. [16]. The detection of genetic diversity among and within cultivars is indispensable for their proper identification, classification and conservation, and mostly for the development of improved genotypes. Our outcomes are particularly important in the context of the proper management of the European chestnut, which is mandatory for the effective exploitation of genetic resources currently threatened by the gall wasp (*Dryocosmus kuriphilus* Y.).

Recent studies in Japanese and European chestnuts evidenced the need for molecular markers more suitable for cost effective and easy-to-use applications [13,23]. In molecular breeding, as well as in other field applications, the request of a high-throughput workflow from DNA isolation to data elaboration has already oriented researchers towards analyses based on SNP markers [11,22,31]. Nonetheless, until now, SNPs were mined within *C. crenata* and interspecific *C. crenata* × *C. sativa* breeding progenies, while information about *C. sativa*-specific SNPs was lacking. HRM analyses evidenced that all 64 selected fragments uniformly distributed among linkage groups in *C. crenata* are also present in *C. sativa* and allowed for the mining of polymorphisms between cultivars with a minimum resequencing effort. In fact, as many as 75% of the investigated loci was indeed polymorphic and yielded, in our resequencing project, 52 completely new SNPs that were useful for varietal discrimination. However, the SNPs originally mined in *C. crenata* were poorly transferable to our genetic pool, as only four of 64 (6.25%) were polymorphic within tested European chestnuts. The molecular tools proposed in the present paper are SNP based, as suggested [11,13,23,31,32] and, as added value, each KASP assay can be analyzed independently from the others. In fact, KASP can be considered perfectly scalable, as a single plate can include from one to 380 samples and, thanks to the standardized cycling protocol, more than a SNP assay can be included if needed [28]. For confirmation, in our analyses, we processed three KASP assays per PCR plate. The proposed molecular recognition tool is reliable and can be used for many cycles/years. In fact, no variability was observed between clones of the same cultivar and this is consistent with the tendential stability of expressed regions during clonal propagation [33]. A higher number of analyzed accessions per cv., possibly coming from different regions of conservation and distribution, would enforce our data. Regarding data management, as shown in Table 2 and Table 3, the gained output is a simple alphabetic code, easy to read, report and query in a database.

The need for the a priori maximization of marker performances oriented us toward allele mining in a set of samples that was representative of the genetic diversity in European chestnut cultivars. A preliminary check of the genetic distance level among genotypes to be tested was carried out by RAPD markers. This technique was preferred because it had already proven useful in the assessment of genetic relatedness for neglected or little-known chestnut cultivars [34,35]. The utility of RAPD was also extensively demonstrated by screening the genetic diversity of different plant species and detecting molecular variations in local populations [36,37]. Our findings are in line with the results of previous studies that utilized RAPD for characterizing the main cultivars of *C. sativa* in the Campania Region [7]. In the present study, RAPD analysis showed that the sampled chestnuts made up a pool of genotypes suitable for mining diversity and for testing the performances of the newly pointed out technologies as being reasonably representative of the range of the genetic diversity degree existing in European chestnut cultivars. In fact, in our variety pool, the distance measured between 0.04–0.27, covering the middle half (65%) of the maximum expected range (from zero to 0.34). Furthermore, the presence in the pool of some clones, as well as of the hybrid ‘Bouche de Bétizac’ ensures that even the extreme expected genetic distances were taken into account.

KASP markers were evaluated as a measure of genetic similarity (or complementary distance) within and between groups of genotypes. These markers are known to be very precise as the high reproducibility of the measurement was previously estimated by Semagn et al. [28], who reported an error smaller than 0.5% when allele calls were manually re-scored. For confirmation, in our results no repeated discording measures were detected. Regarding accuracy, the KASP method successfully evidenced its consistence with RAPD analysis. In fact, for example, the average distance among PCT and NPL genotypes was estimated as 0.06 by RAPD and 0.04 by KASP; the major estimated distance, occurring between ‘Bouche de Bétizac’ and all the other cultivars, was evaluated as 0.29 by RAPD and 0.16 by KASP, with a generally uniform trend. The differences observed were expected, as the polymorphism detected by KASP is related to point mutations occurring on coding regions that are, on average, the less variable regions of the genome. On the contrary, RAPD polymorphisms are often not present in coding sequences, considering that they are associated with repeated and inverted regions of primer binding sites [38]. Moreover, KASP technology is a high-resolution method to measure genetic diversity, provided that the choice of the monitored SNPs is informed and efficient. Being a SNP-based marker, it can indeed measure the smallest possible distance among two genotypes, so that the appropriate SNP can distinguish even between two essentially derived varieties gained from each other by a single point mutation (e.g., by genome editing) [39].

Although the repetitive and complex nature of plant genomes poses a serious obstacle to the researchers interested in developing SNPs [40], this drawback might be overcome by accurate mining for the SNP discovery. The selection of SNPs in non-duplicated genes (avoiding paralogous sequences) might ensure the optimal reliability of SNP detection. Sequences in this study represent low-copy regions, resulting in >90% of the cases in a single, specific match in the genome. Functional description also corroborates that just few genes belong to large gene families (e.g., NAC domain, ERF). For these reasons, the transferability of the proposed SNP is expected to be high between genotypes from different genetic backgrounds, such as *C. sativa* cultivars from different European areas.

Once a panel of SNPs is available, the oriented selection of the assays to be used allows for the complete flexibility of the tool. In fact, a tailored analysis can be performed based on the investigation purpose. For varietal discrimination, the minimum number of required tests can be very low, also depending on the collateral information available. As an example, in our set of genotypes, three SNP assays were enough for distinguishing between nine cultivars. Once a reference database is built, the more the panel of SNP and the reference genotypes are abundant, the less tests are needed. Theoretically, a KASP analysis of 37 SNPs could result in 3^37^ different data strings, corresponding to about half a million trillion different genotypes. Actually, the presence of minor alleles with low frequencies dramatically reduces this value that remains high as well. A broad panel of available assays ensures a good number of available SNPs with high MAF and low heterozygous frequencies that are preferred. For more in-depth analyses, such as phylogeny studies, we can predict that KASP will be a reliable method as soon as a number of SNPs ranging from 112 to 264 are included. In fact, it was reported that the information included in the analyses of a number of microsatellites ranging from 16 to 24 is enough for a robust estimation of distances among European chestnuts [23,41,42] and that, in allogamous species, the variability monitored by a microsatellite corresponds to the variability that can be monitored by seven to 11 SNPs [43]. In the more recent literature, the use of microsatellite molecular markers combined with agronomic and morphological observations was very useful for studying stress adaptation and the genetic origin of the European chestnut populations [20,21,42,44]. The provided widening of the available molecular tools will open up new horizons for these applications.

Our research is ongoing, with more tests involving a larger number of Italian and European cvs, with the objective of building a complete dataset for varietal identification. A wider monitoring of the identified SNPs as well as the enrichment of the available SNP panel could allow us to integrate the European database of chestnut varieties [41] with practical and useful tools for molecular tracking and for commercial purposes. Additional SNPs on cytoplasmic DNA could help traceability systems in processed materials such as chestnut flour, jam, and other derivative foodstuffs. This traceability method could be particularly interesting for regionally recognized product (such as PGI) valorization.

## 4. Materials and Methods

### 4.1. Plant Material and DNA isoLation

Sampling was carried out in the “Roccamonfina—Foce Garigliano” protected area of Region Campania (Italy) in October 2018. Samples from eight cultivars of *C. sativa* and the hybrid cultivar ‘Bouche de Bétizac’, originated by the interspecific hybridization between *C. sativa* cv. ‘Bouche Rouge’ × *C. crenata* selection CA04 [45], were used in this study. Per cultivar, one to three trees were chosen from which to collect leaves from branches of the scion (Table S8). The varietal correspondence of analyzed chestnut cultivars has been preliminarily verified by phenological, agronomic and carpological traits using International Union for the Protection of New Varieties of Plants (UPOV; https://www.upov.int/portal/index.html.en) descriptors. All the trees were georeferenced, photographed and signed by permanent targets for subsequent retrieval. Eight to ten leaves per tree were collected and lyophilized for subsequent analyses. Chestnut genomic DNA was extracted according to the CTAB-based protocol with minor modification by De Masi et al. [36]. DNA isolation for HRM and KASP analyses was performed by DNeasy Plant Mini Kit (Qiagen, Hilden, Germany). DNA concentration and purity were, respectively, checked at 260 nm and by means of 260/280 nm and 260/230 nm ratios in a NanoDrop ND-1000 UV-Vis Spectrophotometer (Thermo Fisher Scientific, Waltham, MA, USA). DNA integrity was visually assessed by agarose gel electrophoresis on random and bulked samples.

### 4.2. Random Amplified Polymorphic DNA (RAPD) Analysis and Clustering

DNA profiling of the chestnut genotypes was performed by RAPD-PCR using the seven arbitrary primers AE19: 5′-GACAGTCCCT-3′, AG14: 5′-CTCTCGGCGA-3′, G12: 5′-CAGCTCACGA-3′, G19: 5′-GTCAGGGCAA-3′, G07: 5′-GAACCTGCGG-3′, U3: 5′-GGGTTTAGGG-3′, U19: 5′-TGGGAACGGT-3′. RAPD amplifications were performed following a previous protocol [37] with some modifications in a reaction volume of 50 μL containing 1X DreamTaq Buffer (Thermo Fisher Scientific, Waltham, MA, USA), 3 mM MgCl_2_, 100 μM each dNTP, 20 pmol of arbitrary primer, 10 ng of chestnut template DNA, and 2.0 Units of DreamTaq™ DNA polymerase (Thermo Fisher Scientific, Waltham, MA, USA). The reactions were carried out in a Veriti 96-Well Thermal Cycler (Applied Biosystems, Foster City, CA, USA) with the following program: initial DNA denaturation for 3 min at 95 °C; 40 amplification cycles each consisting of denaturation for 1 min at 95 °C, primer annealing for 1 min at 40 °C, and primer extension for 1 min at 72 °C; final extension step of 10 min at 72 °C. To verify the reproducibility of the method, PCR negative controls without DNA template were included for each primer, and two independent PCR experiments were performed. RAPD amplicons were separated by 1X TAE buffered 2% agarose gel electrophoresis containing 1X SYBR Safe DNA Gel Stain (Thermo Fisher Scientific, Waltham, MA, USA). Gel banding patterns were visualized under UV light and acquired using ChemiDoc gel documentation system (Bio-Rad, Hercules, CA, USA). Amplicon sizes were estimated using an 1-Kb Plus DNA Ladder as a DNA molecular weight marker (Thermo Fisher Scientific, Waltham, MA, USA). After electrophoresis, RAPD alleles correspondent to reproducible amplicons for each primer were scored as the number of bands per genotype, with “1” for the presence and “0” for the absence of each amplicon based on dominant genetic nature of RAPD markers. The binary data were used to calculate the genetic distance between pairwise genotypes according to Dice’s coefficient (Dc). Dc represents the fraction of RAPD alleles shared between two genotypes, conferring twice the weight to the shared bands. The relationships of the chestnut genotypes were estimated by the triangular matrix of genetic distance data and visualized using a genetic tree-like diagram (dendrogram) constructed using the software PAST (PAleontological STatistics) Ver. 4.01 [46], developed by Hammer et al. [29], according to the unweighted pair group method with the arithmetic mean (UPGMA) clustering algorithm [47]. The Cophenetic Correlation Coefficient (CP) was also calculated to measure the significance of the whole dendrogram. To determine the relevant clusters, a cut-off line was depicted through the UPGMA dendrogram at the Dc mean value.

### 4.3. High-Resolution Melting (HRM) Analysis

For HRM analysis, reactions were built up in a total volume of 15 µL, adding an amount of 7.5 ng of template DNA per reaction in a final concentration of 50 nM of each primer. PrecisionMelt™ EvaGreen^®^ Supermix (Bio-Rad, Hercules, CA, USA) was used according to the manufacturer’s instructions using a CFX96 thermocycler (Bio-Rad, Hercules, CA, USA). A touchdown amplification protocol was used decreasing annealing temperature by 0.5 °C per cycle starting from a temperature 4.5 °C above annealing temperature (Ta, computed as the average between the Tm of the two primers) and for 10 cycles. Cycling was as follows: initial DNA denaturation for 5 min at 95 °C; 40 amplification cycles each consisting of denaturation for 10 s at 95 °C, primer annealing for 15 s as above specified, basing on Tm in Supplementary Table S3, and primer extension for 30 s at 72 °C; a final extension step of 10 min at 72 °C. Melting was conducted after denaturing at 90 °C for 1 min and renaturing PCR products at 50 °C for 2 min. Acquisitions were made during cycling amplification (cycles 13–40), acquiring luminescence for each cycle, and in the denaturation phase, acquiring luminescence for every 0.1 °C. The sizes of amplified fragments were verified by loading some of the products (random and bulked) on agarose gel. Data collection and normalization were performed using the Bio-Rad CFX Manager software; the fluorescence (F) over temperature (T) curve [48] was mainly used. HRM data were analyzed by the Precision Melt Analysis^TM^ v. 1.3 software (Bio-Rad, Hercules, CA, USA). Default settings were used: melt region was detected automatically as well as pre-melt range and post-melt range; these ranges were chosen as small as possible (0.5 °C, corresponding to 5 data points) and as near as possible but not overlapping the melt region. Temperature shift bar height was set to 0.2 and melt curve shape sensitivity for cluster detection was set to 50. The Tm difference threshold for cluster detection was 0.15. Automatically detected clusters per target region were exported in a colored table where, column per column, the same color (and letter) was given to genotypes in the same cluster. Cluster scoring was examined by UPGMA clustering using Hamming index by PAST software [29], and a bootstrapped dendrogram was built recurring to 1000 reiterations. Furthermore, the first 6 principal coordinates computed by the same index in PCoA (representing 74 % of the total variability detected by HRM clustering) were transformed adding the constant value k = 0.50632 and used as independent variables for subsequent linear discriminant analysis (LDA). A canonical variates analysis approach was adopted, where 8 axes were found that best separate the 9 cultivars. The relative confusion matrix was computed.

### 4.4. Target Resequencing

About 184 products previously amplified for HRM analysis were selected for subsequent resequencing, one for each cluster within each fragment preferentially choosing the one with the maximum confidence percentage of cluster attribution (generally 100%). The selected amplified products were purified by adding 15 *µ*moles of ammonium acetate and 2.4 volumes of ethanol and then rinsing once with 70% ethanol. Sanger sequencing of purified products in forward and reverse were performed by an external service in 96-plate format. A total number of 4 to 10 FASTA sequences per target were obtained. Data were then read and edited by SeqScape Software (Version: 1.0, Applied Biosystems). On SeqScape, a reference data group was built up using available sequences for *C. crenata*. Automatically detected polymorphisms were then manually edited and analyzed, evaluating the confidence percentages of base calling and peak quality. All the SNPs were coded, adding to the two letters code of the fragment, three numbers indicating the position of the SNP on the original 161 bp fragment used as reference for the resequencing project. Thirty-seven SNPs were chosen among the SNPs falling in the central part of the fragment, so that at least 50 flanking base pairs were available, and were used for subsequent KASP assay design.

The association of each sequenced fragment with an annotated gene was performed by blasting (e-value threshold 1E-10) the sequences against the transcriptome of *C. sativa* cv. ‘Bouche de Bétizac’ and cv. ‘Madonna’, both retrieved from Aquadro et al. [13]. When no match was found in those transcriptomes, the sequence was blasted against the non-redundant nucleotide collection in NCBI, getting the closest phylogenetic result (mostly *Quercus* and *Fagus*). A functional annotation of the genes was then performed using Blast2GO [49] and gene ontology (GO) terms were predicted by assigning functional classifications [50] as well as the potential properties of gene products. The BLAST cut-off e-value was set to 10^−5^. SNP classification/annotation was assigned according to Ensembl Variation guidelines [51].

### 4.5. Kompetitive Allele-Specific PCR (KASP) Assays

KASP assays were designed by LGC group (London, UK). Analyses were conducted using a CFX96 thermocycler (Bio-Rad, Hercules, CA, USA) in a total reaction volume of 15 µl, where KASP master mix and assays were combined to 7 ng of purified DNA according to the manufacturer’s instructions (LGC group, London, UK). After an initial denaturation at 95 °C for 10 min, a touchdown two-step amplification protocol (20 s at 95 °C and 1 min at the annealing/elongation temperature) was used as recommended, in which the annealing/elongation temperature decreased 0.6 °C per cycle, starting from a temperature of 61 °C for 10 cycles. The acquisition was made at the end after a total of 35 cycles and a step at 37 °C for 1 min, acquiring luminescence in FAM and HEX colors. Where necessary, multiple recycling steps and new data acquisition were made as recommended. The results were analyzed by allelic discrimination tool of the Bio-Rad CFX Manager Software v. 3.1 and exported after automatic allele calling. The resulting allelic table was used for cluster analysis using the DARwin software [52]. Distances were computed by the simple matching index for diploid codominant markers and a dendrogram was built using UPGMA.

## 5. Conclusions

The molecular markers developed here have a high throughput, are ready to use and can be available for identifying cultivars at each step of chestnut cultivation and marketing. They are in line with solutions proposed by other authors that indicated SNP-based markers as reliable and suitable for these purposes. Furthermore, they are scalable, flexible and reliable. The pool of genotypes used for testing their performance was suited to evaluating genetic diversity in European chestnut. The nature of mined diversity, mostly relying on single-copy expressed sequences, suggests that they can be informative even in very different genetic pools and can be steadily associated with each cultivar. The described KASP assays can be a useful and valid tool for the management of chestnut orchards, nurseries and natural parks and can also be used for molecular marker-assisted selection (MAS) in innovative breeding projects. Further studies including more genotypes will allow for reference database implementation and the release of this method to stakeholders.

## Figures and Tables

**Table 1:** Results of resequencing project: per region analyzed, retrieved single nucleotide polymorphisms (SNPs) are reported. The nucleotide triplet harboring the SNP, the amino acid substitution, the variant type, the predicted impact of the variant as well as the functional description and the relative reference sequence were reported.

SNP Code	Description	AA Change	Variant Type	Impact	Functional Description	Ref Sequence_ID
A3079	caC/caT	His/His	synonymous	LOW	uncharacterized protein LOC115978171	Mad_contig_257281
A4081	Aca/Gca	Thr/Ala	missense	MODERATE	two-component response regulator-like APRR9	BdB_contig_30280
A5096	gtA/gtC	Val/Val	synonymous	LOW	uncharacterized membrane protein At3g27390	BdB_contig_81679
A7075	ccA/ccC	Pro/Pro	synonymous	LOW	DExH-box ATP-dependent RNA helicase DExH7	BdB_contig_45109
A8045	caC/caT	His/His	synonymous	LOW	NAC domain-containing protein 35	BdB_contig_49951
A8095	cTg/cAg	Leu/Gln	missense	MODERATE		
A8121	Ttt/Ctt	Phe/Leu	missense	MODERATE		
A9081	ctA/ctT	Leu/Leu	synonymous	LOW	Fanconi anemia group J protein homolog	BdB_contig_161049
B0033	gcA/gcG	Ala/Ala	synonymous	LOW	meiotic recombination protein DMC1 homolog	BdB_contig_344176
B0042	caT/caC	His/His	synonymous	LOW		
B0081	agG/agA	Arg/Arg	synonymous	LOW		
B1077	acA/acG	Thr/Thr	synonymous	LOW	putative E3 ubiquitin-protein ligase RF298	BdB_contig_20383
B2081, B2082	AGa/RRa	Arg/Glu	missense	MODERATE	probable protein phosphatase 2C 42	Mad_contig_4982354
B3127	ctT/ctC	Leu/Leu	synonymous	LOW	nuclear pore complex protein NUP58	XM_024070927
B5060	gaA/gaG	Glu/Glu	missense	MODERATE	trihelix transcription factor GT-2-like	BdB_contig_32135
C0114	agT/agC	Ser/Ser	synonymous	LOW	uncharacterized protein LOC115981485	Mad_contig_4969272
C1083	aAg/aGa	Lys/Arg	missense	MODERATE	intermediate cleaving peptidase 55, mitochondrial	BdB_contig_2198
C1115	atT/atA	Ile/Ile	synonymous	LOW		
C2122	aCt/aAt	-	intergenic	MODIFIER	protein PHR1-LIKE 1-like	XM_024065219
C3057	ggC/ggT	Gly/Gly	synonymous	LOW	pentatricopeptide repeat-containing protein	BdB_contig_73204
C4088	Acc/Gcc	Thr/Ala	missense	MODERATE	uncharacterized protein LOC115978856	BdB_contig_23188
C4092	tTc/tCt	Phe/Ser	missense	MODERATE		
C5126	ccA/ccC	Pro/Pro	synonymous	LOW	peptidyl-prolyl cis-trans isomerase E	XM_024025215
C7034, C7035	cRY/cRY	His/Arg	missense	MODERATE	26S proteasome non-ATPase regulatory subunit 10	XM_024044673
C7040	aAa/aTa	Lys/Ile	missense	MODERATE		
C9098	aCt/aAt	Thr/Asn	missense	MODERATE	dentin sialophospho	BdB_contig_9459
D3098	ggG/ggT	Gly/Gly	synonymous	LOW	Ribosomal protein	BdB_contig_47152
D3104	tcG/tcA	Ser/Ser	synonymous	LOW		
E0104	Ggt/Agt	Gly/Ser	missense	MODERATE	midasin	XM_031113223
E1081	Agg/Ggg	Arg/Gly	missense	MODERATE	probable E3 ubiquitin-protein ligase RHC1A	Mad_contig_9295
E1091	gCt/gGt	Ala/Gly	missense	MODERATE	zinc finger protein ZAT3-like	Mad_contig_9295
E2081	ttT/ttC	Phe/Phe	synonymous	LOW	peroxidase 19	BdB_contig_168068
E2115	cAt/cGt	His/Arg	missense	MODERATE		
F0090	gAa/gGa	Glu/Leu	missense	MODERATE	formin-like protein 5	BdB_contig_78166
F0115	Tat/Cat	Tyr/His	missense	MODERATE		
F0116	ctG/ctT	Leu/Leu	synonymous	LOW		
F1081	tcT/tcA	Ser/Ser	synonymous	LOW	protein-tyrosine-phosphatase IBR5	BdB_contig_59988
F3045	gaC/gaT	Asp/Asp	synonymous	LOW	uncharacterized aarF domain-containing protein kinase	BdB_contig_105211
F3081	Tct/Cct	Ser/Pro	missense	MODERATE		
G0115	Act/Gct	Ala/Thr	missense	MODERATE	probable E3 ubiquitin-protein ligase RHC1A	XM_024060281
G1038	atT/atC	Ile/Ile	synonymous	LOW	protease Do-like 7	BdB_contig_20564
G3081	ggC/ggT	Gly/Gly	synonymous	LOW	phospholipase A-2-activating protein	BdB_contig_6253
G3111	tgT/tgC	Cys/Cys	synonymous	LOW		
G4023	gCg/gTg	Ala/Val	missense	MODERATE	uncharacterized protein LOC112036137	Mad_contig_4962484
G4120	tcT/tcC	Ser/Ser	synonymous	LOW		
G5075	ggG/ggA	Gly/Gly	synonymous	LOW	zinc finger protein ZAT3-like	XM_031086265
G5083	Tcc/Gcc	Ser/Ala	missense	MODERATE		
G5135	ccA/ccG	Pro/Pro	synonymous	LOW		
H0106	tTt/tAt	Phe/Tyr	missense	MODERATE	AP2/ERF and B3 domain-containing RAV1-like	BdB_contig_11865
H2081	gcC/gcT	Ala/Ala	synonymous	LOW	importin-5-like	BdB_contig_11365
H2132	tcG/tcA	Ser/Ser	synonymous	LOW		
H4081	gcC/gcT	Ala/Ala	synonymous	LOW	ATP synthase subunit beta, mitochondrial	BdB_contig_1478
I0081	acG/acA	Thr/Thr	synonymous	LOW	mediator of RNA polymerase II transcription subunit 4	BdB_contig_35713
I2081	gGt/gCt	Gly/Ala	missense	MODERATE	WUSCHEL-related homeobox 11-like	XM_031069633
J0109	cAt/cGt	His/Arg	missense	MODERATE	probable receptor-like serine/threonine-protein kinase	BdB_contig_35898
J1105	tcG/tcC	Ser/Ser	synonymous	LOW	zinc finger CCCH domain-containing protein 12	BdB_contig_42954
J1108	tcT/tcG	Ser/Ser	synonymous	LOW		
J2081	aAc/aGc	Asn/Ser	missense	MODERATE	E3 ubiquitin-protein ligase	BdB_contig_13323
K0044	ggC/ggT	Gly/Gly	synonymous	LOW	nuclear-pore anchor	BdB_contig_7400
K0049	aCa/aTa	Thr/Ile	missense	MODERATE		
K0081	Atg/Gtg/Ttg	Met/Val/Leu	missense	MODERATE		
K1126	atA/atG	Ile/Met	missense	MODERATE	serrate RNA effector molecule-like	BdB_contig_6695
K4081	gaC/gaT	Asp/Asp	synonymous	LOW	O-fucosyltransferase 20	BdB_contig_67709
L0115	gCg/gGg	Ala/Gly	missense	MODERATE	protein DOWNY MILDEW RESISTANCE 6	BdB_contig_72305
L1081	ccG/ccA	Pro/Pro	synonymous	LOW	serine/threonine-protein kinase-like protein	Mad_contig_4983178
L1117	cgA/cgT	Arg/Arg	synonymous	LOW		
L2056	cAt/cGt	His/Arg	missense	MODERATE	uncharacterized protein LOC112025178	BdB_contig_51854
L4081	ctC/ctT	Leu/Leu	synonymous	LOW	GTP cyclohydrolase 1	BdB_contig_65823

**Table 2:** Results of kompetitive allele-specific PCR (KASP) analyses on 20 genotypes of 37 selected SNPs. IUPAC code is used for heterozygous alleles. Question mark for missing data.

Clone	A3079	A5096	A7075	A8095	A9081	B0042	B1077	B2082	B3127	B5060	C0114	C1083	C2122	C3057	C4088	C4092	C9098	D3098	D3104	E0104	E1091	E2115	F1081	F3045	G0115	G4120	G5083	H0106	J0109	J1108	J1105	K0081	K0049	K1126	L0115	L1117	L4081
BdB1	Y	M	T	W	T	Y	R	R	A	G	T	C	C	C	G	Y	A	G	C	C	S	Y	W	R	R	C	M	W	R	G	G	G	T	C	S	A	A
BdB2	Y	M	T	W	T	Y	R	R	A	G	T	C	C	C	G	Y	A	G	C	C	S	Y	W	R	R	C	M	W	R	G	G	G	T	C	S	A	A
BdB3	Y	M	T	W	T	Y	R	R	A	G	T	C	C	C	G	Y	A	G	C	C	S	Y	W	R	R	C	M	W	R	G	G	G	T	C	S	A	A
LCN1	T	A	T	T	A	C	G	A	A	A	T	Y	C	Y	G	C	A	G	C	Y	C	?	A	R	R	Y	M	W	A	G	C	G	C	C	G	A	A
MRZ1	T	M	T	T	A	C	?	R	A	A	Y	Y	C	C	G	C	A	T	Y	C	C	T	A	G	A	T	M	W	A	G	C	R	Y	C	G	W	A
MRZ2	T	M	T	T	A	C	G	R	A	A	Y	Y	C	C	G	C	A	T	Y	C	C	T	A	G	A	T	M	W	A	G	C	R	Y	C	G	W	A
MRC1	Y	M	K	T	T	T	G	G	A	A	Y	C	C	Y	G	C	M	G	C	C	S	Y	W	G	G	Y	M	W	A	K	G	G	Y	Y	G	T	G
MRC2	Y	M	K	T	T	T	G	G	A	A	Y	C	C	Y	G	C	M	G	C	C	S	Y	W	G	G	Y	M	W	A	K	G	G	Y	Y	G	T	G
NPL1	T	A	T	T	W	Y	G	R	A	A	T	T	M	C	G	Y	A	G	C	Y	C	T	A	R	R	Y	A	A	A	G	G	R	Y	Y	G	A	A
NPL2	T	A	T	T	W	Y	G	R	A	A	T	?	M	C	G	Y	A	G	C	Y	C	T	A	R	R	Y	A	A	A	G	G	R	Y	Y	G	A	A
NPL3	T	A	T	T	W	Y	G	R	A	A	T	T	M	C	G	Y	A	G	C	Y	C	T	A	R	R	Y	A	A	A	G	G	R	Y	Y	G	A	A
OLF1	C	C	T	T	A	Y	G	G	A	A	T	Y	C	Y	R	Y	A	G	C	C	C	Y	A	G	A	T	M	A	R	G	G	G	Y	C	G	A	A
PCT1	T	M	T	T	A	Y	G	R	A	A	T	T	M	C	G	Y	A	G	C	Y	C	T	A	R	R	Y	?	W	A	K	C	R	Y	?	G	W	A
PCT2	T	M	T	T	A	Y	G	R	A	A	T	T	M	C	G	Y	A	G	C	Y	C	T	A	R	R	Y	A	W	A	K	C	R	Y	Y	G	W	A
PCT3	T	M	T	T	A	Y	G	R	A	A	T	T	M	C	G	Y	A	G	C	Y	C	T	A	R	R	Y	A	W	A	K	C	R	Y	Y	G	W	A
SPT1	T	A	T	T	A	Y	?	G	R	A	Y	C	C	C	G	Y	A	G	Y	C	C	C	W	G	R	Y	A	A	A	K	G	G	C	T	G	W	R
SPT2	T	A	T	T	A	Y	G	G	R	A	Y	C	C	C	G	?	A	G	Y	C	C	C	W	G	R	Y	A	A	A	K	G	G	C	T	G	W	R
TMP1	T	A	K	T	W	Y	G	R	A	A	T	Y	M	Y	G	Y	A	G	C	Y	C	Y	W	R	G	Y	A	A	R	K	G	G	C	Y	S	A	A
TMP2	T	A	K	T	W	Y	?	R	A	A	T	Y	M	Y	G	Y	A	G	C	Y	C	Y	W	R	G	Y	A	A	R	K	G	G	C	Y	S	A	A
TMP3	T	A	K	T	W	Y	?	R	A	A	T	Y	M	Y	G	Y	A	G	C	Y	C	Y	W	R	G	Y	A	?	R	K	G	G	C	Y	S	A	A

**Table 3:** The three minimum tests required for cv. identification. The unique response code of each cultivar to these tests is reported.

Cultivar	C1083	G0115	A5096
Bouche de Bétizac	C	R	M
Lucente	Y	R	A
Marzatica	Y	A	M
Mercogliana	C	G	M
Napoletana	T	R	A
Olefarella	Y	A	C
Paccuta	T	R	M
San Pietro	C	R	A
Tempestiva	Y	G	A

## Supplementary Materials

Supplementary materials can be found at https://www.mdpi.com/1422-0067/21/13/4805/s1.

## Abbreviations

EST	Expressed Sequence Tag
FRET	Fluorescence Resonance Energy Transfer
HRM	High-Resolution Melting
PGI	Protected Geographical Indication
ISSR	Inter Simple Sequence Repeat
KASP	Kompetitive Allele-Specific PCR
LDA	Linear Discriminant Analysis
MAF	Minor Allele Frequency
MAS	Molecular Marker-Assisted Selection
NCBI	National Center for Biotechnology Information
NGS	Next Generation Sequencing
RAPD	Random Amplified Polymorphic DNA
SNP	Single Nucleotide Polymorphism
SSR	Simple Sequence Repeats
UPGMA	Unweighted Pair Group Method with Arithmetic mean
UPOV	International Union for the Protection of New Varieties of Plants
