# Supplementary material for: Single Nucleotide Polymorphisms as Practical Molecular Tools to Support European Chestnut Agrobiodiversity Management
Source: Int J Mol Sci. 2020 Jul 7;21(13):4805. doi: 10.3390/ijms21134805 (PMC7370276; doi:10.3390/ijms21134805)
Supplement: Supplementary file 1 [file ijms-21-04805-s001.zip › Supplementary files/Table S1 RAPD res.docx]

| **Table S1**. RAPD bands generated using seven arbitrary primers from the nine chestnut genotypes in study. | | | | | | | | |
| --- | --- | --- | --- | --- | --- | --- | --- | --- |
| Genotype * | Private bands | | | | | | | Private bands |
|  | U19 | U3 | AE19 | AG14 | G12 | G19 | G07 |  |
| BdB | - | 2 | 6 | - | 2 | - | - | 10 |
| LCN | - | - | - | - | - | - | - | - |
| MRZ | - | 1 | - | - | - | - | - | 1 |
| MRC | - | - | - | 1 | - | - | - | 1 |
| SPT | - | - | 1 | - | 1 | - | - | 2 |
| NPL | - | - | - | - | - | - | - | - |
| OLF | - | - | - | - | - | 1 | - | 1 |
| PCT | - | - | 1 | - | - | - | - | 1 |
| TMP | - | - | - | - | - | - | - | - |
| Private bands | - | 3 | 8 | 1 | 3 | 1 | - | 16 |
| Polymorphic /total bands | 0/3 | 4/10 | 11/15 | 10/10 | 7/7 | 3/8 | 4/7 | 39/60 |

* BdB: Bouche de Bétizac; LCN: Lucente; MRZ: Marzatica; MRC: Mercogliana; SPT: San Pietro; NPL: Napoletana; OLF: Olefarella; PCT: Paccuta; TMP: Tempestiva.

The sign “-“ was used to indicate not detected bands.
