# Supplementary material for: Single Nucleotide Polymorphisms as Practical Molecular Tools to Support European Chestnut Agrobiodiversity Management
Source: Int J Mol Sci. 2020 Jul 7;21(13):4805. doi: 10.3390/ijms21134805 (PMC7370276; doi:10.3390/ijms21134805)
Supplement: Supplementary file 1 [file ijms-21-04805-s001.zip › Supplementary files/Table S3 Primer HRM.docx]

**Table S3.** List of the primers designed and used for HRM analyses and subsequent fragment resequencing. Name, sequence, length and Tm of each primer are reported. Sub optimal values for product length and flanking regions are underlined.

| **Code^1^** | **Selected fragment^2^** | **Primer Name** | **Primer Sequence (5'->3')** | **Primer length** | **Start^3^** | **Stop^3^** | **Tm** | **Product length** | **# bp flanking left^4^** | **#bp flanking right^4^** | **Net product length^5^** |
| --- | --- | --- | --- | --- | --- | --- | --- | --- | --- | --- | --- |
| A2 | sca02939_15302 | A218F | TTGAGCATGAGCAGCCAAGA | 20 | 3 | 22 | 59.96 | 151 | 59 | 53 | 112 |
|  |  | A218R | GGCTCGAAACTGGTCTGTGA | 20 | 153 | 134 | 59.97 |  |  |  |  |
| A9 | sca02659_6399 | A286F | GGATCGAGCCCAGAGAGAGA | 20 | 6 | 25 | 60.18 | 156 | 56 | 61 | 117 |
|  |  | A289R | GGATTGGGCTTGGAGTGAGT | 20 | 161 | 142 | 59.67 |  |  |  |  |
| A3 | sca02381_25164 | A375F | AATTCCGCCTTTCCAACCCA | 20 | 11 | 30 | 60.18 | 150 | 51 | 58 | 109 |
|  |  | A375R | CAGTAACAAGAAGCAGTAGCGG | 22 | 160 | 139 | 59.33 |  |  |  |  |
| A4 | sca02668_25002 | A483F | AGGATGCAAGTGAAGCCCA | 19 | 1 | 19 | 59.23 | 150 | 62 | 50 | 112 |
|  |  | A483R | ACTGTAGCTCAGCTTGTCCA | 20 | 150 | 131 | 58.66 |  |  |  |  |
| A5 | sca02185_26522 | A559F | GCATCTACATGCTCTTTAAGGGA | 23 | 11 | 33 | 58.29 | 151 | *48* | 62 | 110 |
|  |  | A559R | ATGGAGCCTAACACTGCCC | 19 | 161 | 143 | 59.39 |  |  |  |  |
| A6 | sca06717_19644 | A667F | AGCATCTTCAACAACAATCTTGGG | 24 | 7 | 30 | 60.02 | 152 | 51 | 55 | 106 |
|  |  | A667R | AGAGAATTATCCTGTGCGAAAGT | 23 | 158 | 136 | 57.91 |  |  |  |  |
| A7 | sca02470_29720 | A781F | GCAGGCATATGAACACCTTGC | 21 | 9 | 29 | 60.20 | 153 | 52 | 56 | 108 |
|  |  | A781R | GCAACTGTAGTTTATCTTTGCTGTG | 25 | 161 | 137 | 59.15 |  |  |  |  |
| A8 | sca03514_5640 | A893F | TCAAGTATGATCGGTCAGCAACA | 23 | 1 | 23 | 60.06 | 150 | 58 | 50 | 108 |
|  |  | A893R | CCACAGTCTGTCGGAGAAGG | 20 | 150 | 131 | 59.76 |  |  |  |  |
| B0 | sca03107_23553 | B000F | GGAATGTTCATATCGGATCCAAAG | 24 | 1 | 24 | 57.75 | 153 | 57 | 52 | 109 |
|  |  | B000R | GGATATAGCTTCAGCCTCGGG | 21 | 153 | 133 | 59.79 |  |  |  |  |
| B1 | sca01426_15299 | B121F | AGCTGCTAAATTACGTGCTGC | 21 | 8 | 28 | 59.60 | 154 | 53 | 61 | 114 |
|  |  | B121R | CAGCTGTGTTACTTTGCGTT | 20 | 161 | 142 | 57.24 |  |  |  |  |
| B2 | sca06302_17702 | B255F | AGATGTGGGATGCTTCAGGC | 20 | 6 | 25 | 60.11 | 156 | 56 | 61 | 117 |
|  |  | B255R | ATCGCGGTACCAAAGCAATC | 20 | 161 | 142 | 58.99 |  |  |  |  |
| B3 | sca06512_19062 | B375F | CACAGAGGAAGGGGCAGAAG | 20 | 4 | 23 | 60.04 | 150 | 58 | 53 | 111 |
|  |  | B375R | CCATCGACACAAGTTGCAGG | 20 | 153 | 134 | 59.48 |  |  |  |  |
| B5 | sca07754_6591 | B502F | AGATGACAATGGTGGGAATAATTTG | 25 | 9 | 33 | 57.88 | *145* | *48* | *49* | 97 |
|  |  | B502R | AGTCCTCAGCTTAATCAAGGCTTC | 24 | 153 | 130 | 60.63 |  |  |  |  |
| C0 | sca02520_47630 | C093F | TCCTCAGAGGCACTGGAACA | 20 | 11 | 30 | 60.47 | *146* | 51 | 51 | 102 |
|  |  | C093R | AGCCACATCATATACCATAAGTCCA | 25 | 156 | 132 | 59.45 |  |  |  |  |
| C1 | sca03304_2659 | C185F | GTAGGGCACAACATCTGTCA | 20 | 1 | 20 | 57.53 | 150 | 61 | *48* | 109 |
|  |  | C185R | CAGTTACTGAAAGAAGGGGAGA | 22 | 150 | 129 | 57.11 |  |  |  |  |
| C2 | sca02862_15628 | C280F | TGAAGACATAGCTCCAGCCC | 20 | 10 | 29 | 59.17 | 151 | 52 | 58 | 110 |
|  |  | C280R | GTTGGCAGTGAATTAAGCTTGT | 22 | 160 | 139 | 57.76 |  |  |  |  |
| C3 | sca00033_8948 | C365F | GGCCTTTCACCAAAGGTCGAG | 21 | 7 | 27 | 61.48 | 150 | 54 | 55 | 109 |
|  |  | C365R | GACTCTGCAAGCTGCAAGCAA | 21 | 156 | 136 | 62.01 |  |  |  |  |
| C4 | sca02233_51213 | C440F | TCATTTTTGTTGGGCAACTGGT | 22 | 10 | 31 | 59.50 | 152 | 50 | 61 | 111 |
|  |  | C440R | GGACAAAGGCACGAAGACAA | 20 | 161 | 142 | 58.69 |  |  |  |  |
| C9 | sca07408_2866 | C467F | GAGAAAACATGATCGCAACGGT | 22 | 3 | 24 | 59.84 | 150 | 57 | 52 | 109 |
|  |  | C467R | TCTTGGCTGCTCGAACTTGA | 20 | 152 | 133 | 59.61 |  |  |  |  |
| C5 | sca03640_8319 | C553F | CATCCATGTTGTCCATGGCG | 20 | 5 | 24 | 59.62 | 153 | 57 | 56 | 113 |
|  |  | C553R | ACGAGTCAATACTCCACGCTG | 21 | 157 | 137 | 60.14 |  |  |  |  |
| C7 | sca05007_10636 | C706F | AGGCAGAATGCATCAAAGCAA | 21 | 2 | 22 | 59.10 | 150 | 59 | 51 | 110 |
|  |  | C706R | TCAGCTGAAGCTCGACCAAG | 20 | 151 | 132 | 60.04 |  |  |  |  |
| D0 | sca00704_53668 | D000F | TCCTTGTTGTTGGGGATTTTGA | 22 | 1 | 22 | 58.35 | 150 | 59 | 50 | 109 |
|  |  | D000R | GGTTATGCTGCCCTGGGAAA | 20 | 150 | 131 | 60.32 |  |  |  |  |
| D2 | sca01287_36212 | D200F | TGAGCAACCAAATTTCTGGCTAT | 23 | 5 | 27 | 58.91 | 157 | 54 | 61 | 115 |
|  |  | D200R | GTGGCATCAGACCCTAGTGA | 20 | 161 | 142 | 58.80 |  |  |  |  |
| D3 | sca03918_11002 | D283F | GATTTTGCTGAAAAGGCTCTCA | 22 | 6 | 27 | 57.49 | 150 | 54 | 52 | 106 |
|  |  | D283R | TGAAACAATGCAAAATCTGCACC | 23 | 155 | 133 | 59.19 |  |  |  |  |
| D4 | sca08156_24426 | D402F | TCGTTAGGTTGATCTCCGAGT | 21 | 3 | 23 | 58.28 | 150 | 58 | 52 | 110 |
|  |  | D402R | CTGATAAAGGAAACGCGCCG | 20 | 152 | 133 | 59.97 |  |  |  |  |
| E0 | sca04234_5964 | E061F | CTCCGACCAAGAAAGTGTTCA | 21 | 1 | 21 | 58.17 | 151 | 60 | 50 | 110 |
|  |  | E061R | CTCCAGCTCTTCGTAATCGGT | 21 | 151 | 131 | 59.59 |  |  |  |  |
| E1 | sca06104_13512 | E177F | GCAATGCACTGTTGGAGAGC | 20 | 4 | 23 | 60.11 | 158 | 58 | 60 | 118 |
|  |  | E177R | CTGTGACGGAGAAACATCTGA | 21 | 161 | 141 | 57.70 |  |  |  |  |
| E2 | sca04149_41714 | E267F | GGTCTGTGGCTAGTAAGCCT | 20 | 2 | 21 | 58.80 | 160 | 60 | 62 | 122 |
|  |  | E267R | GCCATTGATCCAAGGCTCC | 19 | 161 | 143 | 58.58 |  |  |  |  |
| E3 | sca06132_16214 | E387F | GCAATGCCGACGGGTTTATG | 20 | 2 | 21 | 60.25 | 152 | 60 | 53 | 113 |
|  |  | E387R | AGTAGTGATCCCCTGCTCCT | 20 | 153 | 134 | 59.36 |  |  |  |  |
| E4 | sca02715_44908 | E478F | GTCTGGCTGAAGTTGGAAGC | 20 | 1 | 20 | 59.12 | 157 | 61 | 57 | 118 |
|  |  | E478R | TTGACCCCTTTGACTTGGGG | 20 | 157 | 138 | 59.81 |  |  |  |  |
| E5 | sca09775_18078 | E532F | TTTTCCAGATAAGACTGACACTGC | 24 | 6 | 29 | 59.00 | 150 | 52 | 52 | 104 |
|  |  | E532R | AACATCACCAAAATACTGTCCCT | 23 | 155 | 133 | 58.00 |  |  |  |  |
| F0 | sca04526_34808 | F019F | AGGAGGTGGTACCTGTCCAT | 20 | 11 | 30 | 59.58 | 151 | 51 | 58 | 109 |
|  |  | F019R | GGATGGCAATTTCCACATTGAGT | 23 | 161 | 139 | 59.80 |  |  |  |  |
| F1 | sca04848_37635 | F139F | TATTCTGAGTCTGGCTGGCT | 20 | 11 | 30 | 58.13 | 151 | 51 | 60 | 111 |
|  |  | F139R | CCTCCTTCGTTTAGCTTTGGC | 21 | 161 | 141 | 59.53 |  |  |  |  |
| F2 | sca02428_5565 | F243F | TCTGAATCCAAACATTAGTCAGCAC | 25 | 5 | 29 | 59.59 | 151 | 52 | 50 | 102 |
|  |  | F243R | ACAAAGCAAAGAACAAGAACAAAGG | 25 | 155 | 131 | 59.36 |  |  |  |  |
| F3 | sca00446_18597 | F348F | AGATGAGGCCTGCAACCAAA | 20 | 4 | 23 | 59.89 | 158 | 58 | 60 | 118 |
|  |  | F348R | ACAGAATTGTCTGGTGTGGAT | 21 | 161 | 141 | 57.20 |  |  |  |  |
| F4 | sca01138_80330 | F453F | TCCTCTCTTGACTTGGCAGA | 20 | 1 | 20 | 57.98 | 153 | 61 | 54 | 115 |
|  |  | F453R | TCAGGAGTCTCTTGCCCGA | 19 | 153 | 135 | 59.92 |  |  |  |  |
| F5 | sca03035_5778 | F545F | GTGCAGCGATGGGAGAGAAA | 20 | 10 | 29 | 60.39 | 151 | 52 | 57 | 109 |
|  |  | F545R | ACCACAGCATGTACTCATTCTCA | 23 | 160 | 138 | 59.74 |  |  |  |  |
| G0 | sca00602_82856 | G000F | TCCAGTCACTCGACACCTTG | 20 | 5 | 24 | 59.33 | 151 | 57 | 53 | 110 |
|  |  | G000R | TGGAGGGTGATCCAAGATTGTT | 22 | 155 | 134 | 59.35 |  |  |  |  |
| G1 | sca05047_19856 | G106F | TGATCTGTATGATTCCAGGGAC | 22 | 9 | 30 | 57.04 | 150 | 51 | 56 | 107 |
|  |  | G106R | TTCAGGAAGAAATCCAAGAGCA | 22 | 158 | 137 | 57.63 |  |  |  |  |
| G2 | sca09572_16899 | G226F | TCTCACTTGTATTTGGCGAGCAA | 23 | 1 | 23 | 60.81 | 154 | 58 | 50 | 108 |
|  |  | G226R | ACTTTTTGGTGTCTTTCTTGTGTG | 24 | 154 | 131 | 58.53 |  |  |  |  |
| G3 | sca03637_28269 | G367F | TTCCAGTGAGTCAGCCAACC | 20 | 3 | 22 | 59.89 | 150 | 59 | *49* | 108 |
|  |  | G367R | TCAGATGGAGTTTGTGTTCAGAC | 23 | 152 | 130 | 58.62 |  |  |  |  |
| G4 | sca01457_1084 | G463F | CCGCCGTCGGCCATAAC | 17 | 1 | 17 | 60.98 | 154 | 64 | 55 | 119 |
|  |  | G463R | CGAAAATGCCCTTTCGCCG | 19 | 154 | 136 | 60.51 |  |  |  |  |
| G5 | sca01748_33399 | G553F | GCCGCCATAGACTCCCTAAAA | 21 | 1 | 21 | 59.86 | 154 | 60 | 57 | 117 |
|  |  | G553R | CGGTGGCGGTTATGGCA | 17 | 154 | 138 | 60.09 |  |  |  |  |
| H0 | sca07852_26093 | H025F | AAAGCTGTTCACGCGCTTTC | 20 | 8 | 27 | 60.32 | 154 | 54 | 60 | 114 |
|  |  | H025R | GACATGTTGAGGAAACACACG | 21 | 161 | 141 | 57.75 |  |  |  |  |
| H1 | Sca00112_16392 | H151F | TTGGACGGGGACGTTAAAACT | 21 | 1 | 21 | 59.86 | 151 | 60 | 56 | 116 |
|  |  | H151R | TCTCCCTTCCCTGTGCACTC | 20 | 156 | 137 | 61.19 |  |  |  |  |
| H2 | sca07772_277 | H267F | GCATCTGTTATGCAGGTGGC | 20 | 7 | 26 | 59.62 | 151 | 55 | 52 | 107 |
|  |  | H267R | AGAGATTTAGCAAGAATTGGAATGT | 25 | 157 | 133 | 57.05 |  |  |  |  |
| H4 | sca04049_28022 | H423F | CCTCCAAGATCCGTAGCCAA | 20 | 1 | 20 | 59.17 | 161 | 61 | 51 | 112 |
|  |  | H423R | ACCCAACATCATGCGATTCT | 20 | 151 | 132 | 57.57 |  |  |  |  |
| I0 | sca05845_24767 | I001F | TCTTGTACTACCACTGTTGCATCT | 24 | 7 | 30 | 59.72 | 154 | 51 | 60 | 111 |
|  |  | I001R | TTTGCTCTTCTTGTGGCGCA | 20 | 160 | 141 | 61.10 |  |  |  |  |
| I1 | sca01095_74116 | I118F | AGCATTGAGTCTGAAGTGAGTG | 22 | 4 | 25 | 58.34 | 156 | 56 | 54 | 110 |
|  |  | I118R | TTCTGTGACTTTAACTTCCAGTTCC | 25 | 159 | 135 | 59.18 |  |  |  |  |
| I2 | sca01520_45323 | I245F | TGCAAATTTGAAGTATCGGAAGC | 23 | 3 | 25 | 58.27 | 156 | 56 | 56 | 112 |
|  |  | I245R | TCTGGGTTCCTCTTCTTCTTCA | 22 | 158 | 137 | 58.41 |  |  |  |  |
| I3 | sca09253_1684 | I339F | TCAATGTTGAAACCAATCGTGATA | 24 | 3 | 26 | 57.19 | *145* | 55 | *48* | 103 |
|  |  | I339R | TGCAAGCTTGAAAGAGCCA | 19 | 147 | 129 | 57.89 |  |  |  |  |
| J0 | sca00506_37802 | J090F | AGAGTGAGTCATCCCCCACA | 20 | 8 | 27 | 59.88 | 150 | 54 | 52 | 106 |
|  |  | J090R | TTCAAGGTGATATTGAGGTCACCAA | 25 | 157 | 133 | 60.22 |  |  |  |  |
| J1 | sca07695_14795 | J168F | GTTACTAACTGTAATTTCGCTCACA | 25 | 4 | 28 | 57.66 | 150 | 53 | 52 | 105 |
|  |  | J168R | ATTGGATGAGCCCAATGAGGG | 21 | 153 | 133 | 60.13 |  |  |  |  |
| J2 | sca00093_34686 | J281F | TTTTTCCACAAAGAGTGTGTCG | 22 | 2 | 23 | 57.65 | 158 | 58 | 58 | 116 |
|  |  | J281R | GCTCACCAAGCCATTCCCTAT | 21 | 159 | 139 | 60.13 |  |  |  |  |
| J3 | sca09469_16130 | J391F | TCGGTAAGCTTAAGCACCCA | 20 | 6 | 25 | 59.02 | 150 | 56 | 55 | 111 |
|  |  | J391R | TCGCGATTTTACGCCTTCTT | 20 | 155 | 136 | 58.28 |  |  |  |  |
| J5 | sca01197_40063 | J523F | CTGAAGGCCACCTGCAAATC | 20 | 9 | 28 | 59.47 | 150 | 53 | 57 | 110 |
|  |  | J523R | GTCCCTGTCGAAGAGGAAAGG | 21 | 158 | 138 | 60.07 |  |  |  |  |
| K0 | sca00797_47756 | K017F | GGCCACGCCTTGTAAGACC | 19 | 10 | 28 | 61.04 | 151 | 53 | 58 | 111 |
|  |  | K017R | CGTTTGCGGACTAATGGTTGAG | 22 | 160 | 139 | 60.16 |  |  |  |  |
| K1 | sca09540_14535 | K149F | AAGCTTTAAGTCTTTCAGAACACGA | 25 | 2 | 26 | 59.18 | 150 | 55 | 50 | 105 |
|  |  | K149R | ATGATGCGTTCAGTGCACAAG | 21 | 151 | 131 | 59.80 |  |  |  |  |
| K2 | sca03324_38271 | K206F | AGGTCAAAGTTTTTGTATGGCTCA | 24 | 1 | 24 | 59.11 | 160 | 57 | 56 | 113 |
|  |  | K206R | CTGCACTAGAGATAACATTTGCAT | 24 | 160 | 137 | 57.38 |  |  |  |  |
| K4 | sca00210_23950 | K403F | GCAATGACCACAGCATCCAC | 20 | 1 | 20 | 59.83 | 156 | 61 | 56 | 117 |
|  |  | K403R | AGATGGGTTGGGGTACAAGC | 20 | 156 | 137 | 59.67 |  |  |  |  |
| L0 | sca02390_42028 | L109F | AGCATTTGGGTGTGGATTGAC | 21 | 6 | 26 | 59.10 | 150 | 55 | 56 | 111 |
|  |  | L109R | CCACCATGTCCGGAACCAG | 19 | 155 | 137 | 60.38 |  |  |  |  |
| L1 | sca03738_22943 | L196F | AGCGATGTTTTCAGCTTCGG | 20 | 1 | 20 | 59.20 | 157 | 61 | 58 | 119 |
|  |  | L196R | GAGATCCGACCCGAACGTC | 19 | 157 | 139 | 59.94 |  |  |  |  |
| L2 | sca03650_42786 | L271F | AGTTTGAAAATGAGGTGGTAGAAGT | 25 | 5 | 29 | 58.63 | 151 | 52 | 52 | 104 |
|  |  | L271R | TCCACATCCTCAAAGAGAATCAA | 23 | 155 | 133 | 57.50 |  |  |  |  |
| L4 | sca01010_949 | L426F | ACCAGAAGGGACATAACCCAC | 21 | 6 | 26 | 59.37 | 150 | 55 | 55 | 110 |
|  |  | L426R | TTCCCTGAAGCTGGTCTGGA | 20 | 155 | 136 | 60.47 |  |  |  |  |

^1^ The two-letter code corresponds to the approximate position of the fragment on the linkage maps of *C. crenata*

^2^ Code of the corresponding 161 bp fragment by Nishio et al. (2018)

^3^ The coordinates of the extremes (start and stop) of the annealing region of each primer referred to the 161bp corresponding fragment

^4^ The number of bp of the regions flanking the putative SNP position are computed from the SNP position (81) to the primer annealing region

^5^ Net product length indicates the length of the product between the primer annealing regions.
