# Supplementary material for: Single Nucleotide Polymorphisms as Practical Molecular Tools to Support European Chestnut Agrobiodiversity Management
Source: Int J Mol Sci. 2020 Jul 7;21(13):4805. doi: 10.3390/ijms21134805 (PMC7370276; doi:10.3390/ijms21134805)
Supplement: Supplementary file 1 [file ijms-21-04805-s001.zip › Supplementary files/Table S5 Confusion Matrix.docx]

**Table S5.** Confusion matrix.

| **Cultivar^1^** | **BdB** | **LCN** | **MRZ** | **MRC** | **NPL** | **OLF** | **PCT** | **SPT** | **TMP** | **Total** |
| --- | --- | --- | --- | --- | --- | --- | --- | --- | --- | --- |
| BdB | 3 | 0 | 0 | 0 | 0 | 0 | 0 | 0 | 0 | 3 |
| LCN | 0 | 1 | 0 | 0 | 0 | 0 | 0 | 0 | 0 | 1 |
| MRZ | 0 | 0 | 2 | 0 | 0 | 0 | 0 | 0 | 0 | 2 |
| MRC | 0 | 0 | 0 | 2 | 0 | 0 | 0 | 0 | 0 | 2 |
| NPL | 0 | 0 | 0 | 0 | 3 | 0 | 0 | 0 | 0 | 3 |
| OLF | 0 | 0 | 0 | 0 | 0 | 1 | 0 | 0 | 0 | 1 |
| PCT | 0 | 0 | 0 | 0 | **1** | 0 | 2 | 0 | 0 | 3 |
| SPT | 0 | 0 | 0 | 0 | 0 | 0 | 0 | 2 | 0 | 2 |
| TMP | 0 | 0 | 0 | 0 | 0 | 0 | 0 | 0 | 3 | 3 |
| Total | 3 | 1 | 2 | 2 | 4 | 1 | 2 | 2 | 3 | 20 |

^1^On the first column, the correct cultivar name; on the first raw, the cultivar attribution based on LDA. Attribution was correct in 95% of the times
