# Supplementary material for: Single Nucleotide Polymorphisms as Practical Molecular Tools to Support European Chestnut Agrobiodiversity Management
Source: Int J Mol Sci. 2020 Jul 7;21(13):4805. doi: 10.3390/ijms21134805 (PMC7370276; doi:10.3390/ijms21134805)
Supplement: Supplementary file 1 [file ijms-21-04805-s001.zip › Supplementary files/Table S8 Plant Mat.docx]

**Table S8.** Plant material.

| **Cultivar** | **Cultivar synonymous** | **Clone** | **Latitude** | **Longitude** |
| --- | --- | --- | --- | --- |
| Bouche de Bétizac | - | BdB1 | 41.27445 | 13.96937 |
| Bouche de Bétizac | - | BdB2 | 41.27453 | 13.96916 |
| Bouche de Bétizac | - | BdB3 | 41.27451 | 13.96912 |
| Luciente | Lucida | LCN1 | 41.27452 | 13.97000 |
| Marzatica | Marzara, Mazzara | MRZ1 | 41.29542 | 14.01231 |
| Marzatica | Marzara, Mazzara | MRZ2 | 41.29499 | 14.01185 |
| Mercogliana | Merculiana | MRC1 | 41.27535 | 13.97022 |
| Mercogliana | Merculiana | MRC2 | 41.27522 | 13.97074 |
| Napoletana | Riccia Napoletana | NPL1 | 41.27527 | 13.97010 |
| Napoletana | Riccia Napoletana | NPL2 | 41.27519 | 13.97022 |
| Napoletana | Riccia Napoletana | NPL3 | 41.27509 | 13.97039 |
| Ulefarella | Olefarella | OLF1 | 41.28726 | 13.99205 |
| Pacona | Paccuta | PCT1 | 41.31232 | 13.97400 |
| Pacona | Paccuta | PCT2 | 41.31192 | 13.97379 |
| Pacona | Paccuta | PCT3 | 41.31189 | 13.97400 |
| San Pietro | - | SPT1 | 41.29528 | 14.01213 |
| San Pietro | - | SPT2 | 41.29548 | 14.01237 |
| Tempestiva | Precoce di Roccamonfina, Primitiva | TMP1 | 41.28671 | 13.97759 |
| Tempestiva | Precoce di Roccamonfina, Primitiva | TMP2 | 41.28683 | 13.97763 |
| Tempestiva | Precoce di Roccamonfina, Primitiva | TMP3 | 41.28674 | 13.97751 |
