# Supplementary figures and images for: Single Nucleotide Polymorphisms as Practical Molecular Tools to Support European Chestnut Agrobiodiversity Management
Source: Int J Mol Sci. 2020 Jul 7;21(13):4805. doi: 10.3390/ijms21134805 (PMC7370276; doi:10.3390/ijms21134805)

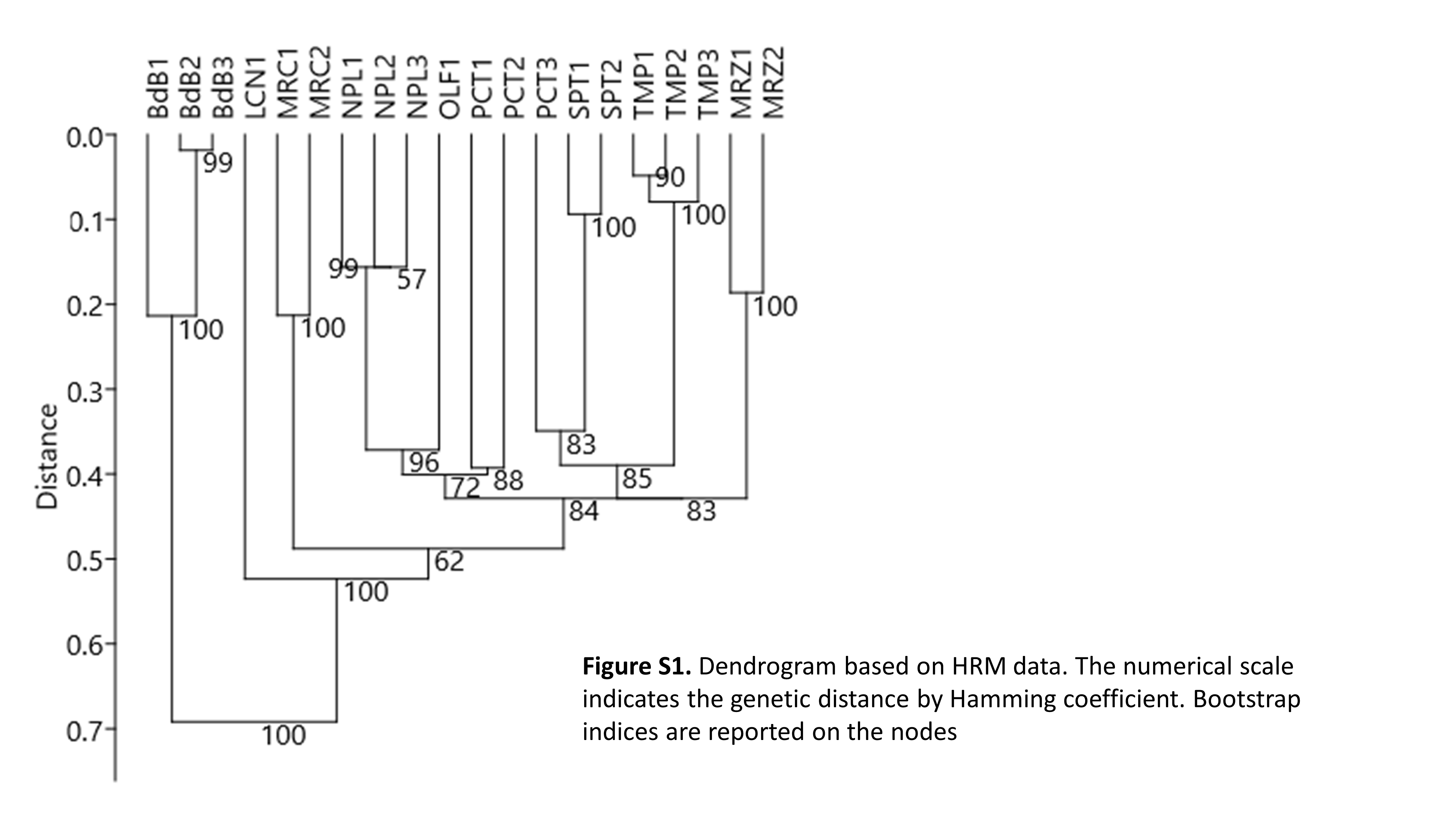

Supplement: Supplementary file 1 [file ijms-21-04805-s001.zip › Supplementary files/Figure S1.tif]
